# Diagnostic Value of Artificial Intelligence Based on CT Image in Benign and Malignant Pulmonary Nodules

**DOI:** 10.1155/2022/5818423

**Published:** 2022-03-24

**Authors:** Wang Du, Bei He, Xiaojie Luo, Min Chen

**Affiliations:** Department of Radiology, Beijing Hospital, National Center of Gerontology, Institute of Geriatric Medicine, Chinese Academy of Medical Science, Beijing, China

## Abstract

**Objective:**

To evaluate the diagnostic value of artificial intelligence-assisted CT imaging in benign and malignant pulmonary nodules.

**Methods:**

The CT scan screening of pulmonary nodules from November 2018 to November 2020 was retrospectively collected. The diagnosis of pulmonary nodules and surgical treatment were performed. A total of 194 nodules in 152 patients with clear pathological results were observed. All patients underwent CT examination to analyze the consistency of the results of artificial intelligence, physician reading according to imaging features, multidisciplinary team work (MDT) diagnosis, and postoperative pathological results; the diagnostic efficacy of different diagnostic methods for solitary pulmonary nodules and the differences of ROC curve and AUC were analyzed. The accuracy, specificity, sensitivity, positive predictive value, negative predictive value, false negative rate, and false positive rate of different diagnostic methods for pulmonary nodules were calculated, and the ROC curves of different diagnostic methods were plotted.

**Results:**

The accuracy, sensitivity, specificity, and Youden index of artificial intelligence (AI) were 89.69%, 92.98%, 65.22%, and 58.20%; the accuracy, sensitivity, specificity, and Youden index of physician reading were 85.57%, 88.30%, 65.22%, and 53.52%; the accuracy, sensitivity, specificity, and Youden index of MDT were 96.91%, 98.25%, 86.96%, and 85.21%, respectively. The kappa values of artificial intelligence, physician reading, and MDT were 0.541, 0.437, and 0.852, and the AUC was 0.768, 0.791, and 0.926, respectively (*P* < 0.001). The average detection time of pulmonary nodules in the AI group, manual reading group, and MAT group was (145 ± 97) s, (534 ± 297) s, and (421 ± 128) s (*P* < 0.001).

**Conclusion:**

Artificial intelligence pulmonary nodule detection system can improve the coincidence rate and accuracy of early diagnosis of lung cancer, shorten the average detection time, and provide more accurate information for clinical decision-making.

## 1. Introduction

The incidence and mortality of lung cancer are the highest in all malignant tumors, and the incidence is increasing year by year [1, 2]. The 5-year survival rate of patients with advanced lung cancer was only 18%, while the 5-year survival rate of early lung cancer after treatment can reach more than 90% [3]. However, patients with early lung cancer usually have no symptoms and are difficult to detect, and most of them are in the advanced stages once clinical symptoms occur. Therefore, improving the cure rate and prognosis of patients with lung cancer depends on early diagnosis and early treatment [4]. The effective way to move forward the diagnostic port of lung cancer is early screening. Chest CT scan has high-density resolution, and it is considered to be one of the most mature and effective imaging techniques for early screening of lung cancer [5, 6].

Early lung cancer is mainly manifested as pulmonary nodules (ground glass nodules, mixed nodules, and solid nodules) on CT images. How to accurately determine the benign and malignant pulmonary nodules in a noninvasive method is a difficult and hot spot of current research [7]. Because of the comprehensive popularization of thin-slice CT, very small pulmonary nodules can be screened. At the same time, because of the increase in the number of scanning slices, imaging physicians are faced with huge data and images, which is not only time-consuming and laborious, low work efficiency, but also inefficient and easy to cause misdiagnosis or missed diagnosis [8]. Therefore, the traditional manual reading has been unable to meet the clinical needs.

Computer-aided diagnosis (CAD) refers to the computer-aided technology that combines imaging, medical image processing, and other technologies with the strong computational analysis capabilities of the computer to assist physicians finding lesions, providing effective auxiliary diagnostic information, reducing the work burden, and improving the work efficiency and diagnostic accuracy [9]. In recent years, with the continuous development of CAD technology, it is possible to use artificial intelligence (AI) technology to screen massive CT images and mark suspicious lesions.

In our study, the postoperative pathological results of pulmonary nodules were used as the gold standard for diagnosis, and the value of artificial intelligence (AI) in CT screening of pulmonary nodules was studied compared with physician reading, imaging characteristics, and multidisciplinary teamwork (MDT) diagnostic opinions.

## 2. Subjects and Methods

### 2.1. Subjects

A retrospective analysis of 5248 cases of physical examination who underwent CT scanning screening for pulmonary nodules from November 2018 to November 2020 was conducted. In addition, 72 cases with 97 nodules diagnosed as pulmonary nodules and confirmed by surgery and pathology were selected as the observation subjects. This study was approved by the Ethics Committee of Bei Jing Hospital.

The inclusion criteria were as follows: according to the Chinese Expert Consensus on Diagnosis and Treatment of Pulmonary Nodules (2018), patients were diagnosed as having pulmonary nodules; pulmonary nodules are highly suspected or cannot rule out the possibility of malignancy, or patients with extreme anxiety affect normal life, and surgical treatment is urgently required; pulmonary nodule resection was performed in the hospital, and the postoperative histopathological results were confirmed to be known after examination by the pathology department; artificial intelligence analysis of chest CT was performed in the hospital before operation.

The exclusion criteria were as follows: have a clear history of malignant tumors, or have undergone surgery, chemoradiotherapy, and other treatments; the size of pulmonary nodules is greater than 3 cm, or accompanied by significant hilar enlargement, atelectasis, and pleural effusion; and patients with imperfect records of chest CT imaging features or MDT diagnostic opinions.

### 2.2. Multislice Spiral CT Examination Method

The chest CT image was obtained with equipment from different manufacturers by using standard imaging protocols. Chest CT scan field from the thoracic inlet to the base of the lungs. Protocols are as follows: scanning slice thickness, 5 mm; image matrix, 512 × 512; FOV, 400 mm; reconstruction parameter setting: slice thickness, 1.0 mm; layer spacing, 1.0 mm; observation window parameters: lung window width, 1500 HU; window position, −600 HU; mediastinum window width, 400HU; window level, 35 HU.

### 2.3. Artificial Intelligence Analysis and Diagnosis Method

Pulmonary nodules were analyzed and diagnosed based on artificial intelligence products. Through the analysis on the images by the system after uploading the chest CT images of the patients, the location, size, volume, density, and malignant probability of suspected pulmonary nodules are finally accurately displayed; if the probability is >70%, it suggests that the nodule is diagnosed as a malignant nodule.

### 2.4. Physician Reading Diagnostic Method

The malignant signs in high-resolution CT images of the patient's chest, such as ground glass nodule, hairpin sign, vascular sign, bronchial sign, lobulation sign, bubble shadow, and so on, were used as the basis of diagnosis. The diagnosis of the risk degree of pulmonary nodules was given after comprehensive consideration by two professional physicians in the imaging department of the hospital with more than 5 years of work experience, and the diagnosis was malignant if high risk was considered.

### 2.5. Multidisciplinary Teamwork (MDT) Diagnostic Method

The multidisciplinary collaborative diagnosis and treatment team was composed of 3 experts from the Department of Respiratory Surgery, the Department of Thoracic Surgery, and the Department of Imaging of the hospital. The consultation was performed in the multidisciplinary consultation center. The risk degree of pulmonary nodules was diagnosed according to the clinical basic situation, relevant auxiliary examination results, and their dynamic changes. The high risk of diagnosis was considered malignant.

### 2.6. Pathological Diagnosis Method

The specimens of pulmonary nodules obtained after pulmonary nodule resection in the Department of Thoracic Surgery of the hospital were all subjected to pathological examination in the Department of Pathology of the hospital. Through formalin fixation, paraffin embedding and sectioning, staining with special methods, and other steps, the final pathological diagnosis is based on the 2015 WHO pathological classification system, and the final pathological diagnosis was determined by two or more chief physicians or a deputy director and above.

### 2.7. Data Collection and Analysis

Basic information such as gender and age of the study subjects was collected. High-resolution CT imaging characteristics of the chest (ground glass-like nodules, burr sign, vascular sign, bronchial sign, lobulation sign, and bubble shadow), the results of artificial intelligence analysis of chest CT, and postoperative pathological results were collected. The consistency between the diagnosis results of artificial intelligence, film reading by physicians based on imaging features, and MDT diagnosis with postoperative pathological results was analyzed. To assess the diagnostic efficacy of different diagnostic methods alone in the diagnosis of pulmonary nodules, the differences in ROC curve and AUC were analyzed.

### 2.8. Statistical Analysis

IBM SPSS 24.0 was used for statistical analysis, and measurement data in line with normal distribution are represented as mean ± standard deviation. Data are counted using the kappa test for the data consistency test; kappa > 0.4 represents consistency. The accuracy, specificity, sensitivity, positive predictive value, negative predictive value, false negative rate, and false positive rate of different diagnostic methods for pulmonary nodules were calculated. The ROC curves of different diagnostic methods were drawn. The AUC >0.7 represents a good diagnostic value. *P* < 0.05 was considered statistically significant.

## 3. Results

### 3.1. Basic Information

There were 152 patients confirmed by pathology; 71 were male and 81 were female, age ranged from 30 to 87 years, with an average age of 57.1 ± 8.12 years. There were 39 patients with solitary nodules and 113 patients with multiple nodules, of which 194 nodules were confirmed by surgery and pathology, and 171 malignant nodules (18 poorly differentiated carcinomas, 27 atypical adenomatous hyperplasia, 39 adenocarcinoma in situ, 48 microinvasive adenocarcinomas, and 39 invasive adenocarcinomas) and 23 benign nodules (9 sarcoidosis, 7 inflammatory granulomas, and 7 tuberculoma) were pathologically diagnosed as lung cancer (Table 1).

### 3.2. Consistency Analysis between Three Diagnostic Methods and Pathological Results

Among the 167 pulmonary nodules suggested by AI, 159 were pathologically confirmed as malignant and 8 as benign; of the 27 pulmonary nodules suggested by AI as intermediate and low risk, 12 were pathologically confirmed as malignant and 15 as benign. Among the 159 pulmonary nodules diagnosed as malignant by physicians, 151 were pathologically confirmed to be malignant and 8 were benign. Among 35 low-risk pulmonary nodules suggested by physician reading diagnosis, 20 were pathologically confirmed to be malignant and 15 were benign. Among the 171 pulmonary nodules with high risk suggested by MDT diagnosis, 168 were pathologically confirmed as malignant and 3 were benign. Among the 23 pulmonary nodules with medium and low risk suggested by MDT diagnosis, 3 were pathologically confirmed as malignant and 20 were benign.

The kappa values for AI, physician reading, and MDT were 0.541, 0.437, and 0.852, respectively (Table 2).

### 3.3. Analysis of Diagnostic Efficacy of Three Diagnostic Methods

The accuracy of artificial intelligence (AI) diagnosis was 89.69%, sensitivity was 92.98%, and specificity was 65.22%; the accuracy of physician reading diagnosis was 85.57%, sensitivity was 88.30%, and specificity was 65.22%; and the accuracy of MDT diagnosis was 96.91%, sensitivity was 98.25%, and specificity was 86.96%.

The false negative rates of the three diagnostic methods of artificial intelligence, physician reading, and MDT were 7.02%, 11.70%, and 1.75%; the false positive rates were 34.78%, 34.78%, and 13.04%; and the Youden indices were 58.20%, 53.52%, and 85.21%, respectively (Table 3).

### 3.4. Receiver Operating Characteristic Curves for Three Diagnostic Methods

**Figure 1:**
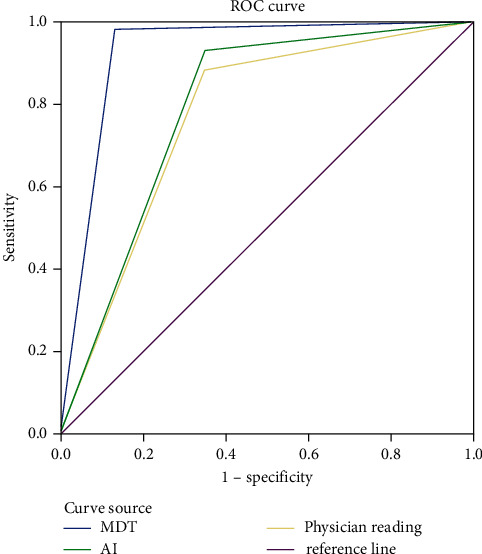
ROC curve of three diagnostic methods for malignant pulmonary nodules.

The AUCs of artificial intelligence, physician reading, and MDT were 0.768, 0.791, and 0.926, respectively. The AUCs of the three diagnostic methods were >0.7, indicating that the three diagnostic methods all had good diagnostic value (Table 4 and Figure 1).

### 3.5. Comparison of Detection Time of Three Diagnostic Methods

Because of the complexity of imaging analysis and the difficulty of nodule diagnosis, the reading time of physicians for each screener was very different, and the average time for analyzing pulmonary nodules is (534 ± 297) s. In addition, with the increase of reading amount and time, the reading time will be significantly increased. When there are 50 consecutive readings, the average time to detect pulmonary nodules increased to (724 ± 1317) s. However, the average time of detecting pulmonary nodules in the AI group was (145 ± 97) s. With the increase of reading, there was no significant difference in the average time of detecting pulmonary nodules. In addition, MAT group time is less than the doctor reading group. The difference in detection time among the three diagnostic methods was statistically significant (*P* ≤ 0.001) (Table 5).

## 4. Discussion

Because of the high incidence and mortality of malignant lung cancer, more and more attention has been paid to its early diagnosis and treatment, and as an important early marker, the differentiation of its nature has been the focus and difficulty in clinical work [10]. In recent years, epidemiological studies have shown that the incidence and mortality of lung cancer are increasing [11]. With the gradual popularization of early lung cancer screening in the population and the detection rate of pulmonary nodules greatly increased, it further increases the challenges for its nature evaluation and identification in clinical work.

In addition, the use of low-dose computed tomography (LDCT) for screening high-risk population to improve the diagnostic effect and prognosis of early lung cancer has become the consensus of many international authoritative medical organizations [12]. CT has high sensitivity in the detection of pulmonary nodules and early lung cancer. Early detection of pulmonary nodules by low-dose CT is very important for the diagnosis and treatment of lung cancer. However, the analysis of a large number of CT images is also a challenge for radiologists, especially for large-scale screening of high-risk population, fatigue caused by a large number of image reading, and other factors, which make false detection and missed detection almost inevitable. Because of the advantages of self-learning images and feature extraction under the conditions of given tasks, AI-assisted diagnostic system can reduce the manual participation in the diagnostic process, avoid the subjective deviation, and improve the efficiency and objectivity of analysis. At present, it has been increasingly used in clinical practice in the field of diagnosis of pulmonary nodules, providing help for clinicians, and its diagnostic value has also been greatly recognized [13].

In our study, the pathological results after surgery were used as the gold standard for the diagnosis of pulmonary nodules, and compared with the diagnostic results of AI, the kappa value was 0.541, indicating that the two methods were consistent. The accuracy, sensitivity, specificity, and Youden index of artificial intelligence diagnostic results were 89.69%, 92.98%, 65.22%, and 58.20%, respectively. Compared with the results of physician reading diagnosis (accuracy 85.57%, sensitivity 88.30%, specificity 65.22%, and Youden index 53.52%), it showed that artificial intelligence had better diagnostic efficacy. However, there was also a high false positive rate (34.78%) in the AI-assisted diagnostic system. The reasons may be as follows: first, since the artificial intelligence system based on deep learning is diagnosed by the implicit features in the data marked by a large number of autonomous learning data, it is difficult to clearly explain the reasons for its good results and it is also difficult to make targeted improvements when errors occur. Second, the diagnosis of artificial intelligence depends on the training of a large number of diverse data and its diagnostic level is directly related to the diversity of the quantity, quality, and distribution level of training data, but it is difficult to sample the data of all populations in the actual situation, which inevitably leads to certain sampling bias of training data. Third, in clinical practice, patients may often have other lung diseases, such as vascular cross-section (especially in hilar vessels), first costal cartilage calcification, interlobular septal thickening, vascular thickening, and bronchial mucus plug; scars and localized pleural thickening caused by various chronic inflammations may be misdiagnosed as nodules by the AI-assisted diagnostic system. This may have a certain impact on the efficacy of artificial intelligence diagnosis, resulting in the instability of its diagnostic ability and the problems of misdiagnosis and missed diagnosis. Therefore, although artificial intelligence has good diagnostic value, it still cannot completely replace the postoperative pathological results as the gold standard for the diagnosis of the nature of pulmonary nodules. The missed diagnosis rate (11.70%) and misdiagnosis rate (34.78%) of double reading by imaging physicians were higher than those of the other two examination methods. The main reason is that some nodules are small pulmonary nodules<5 mm, which are difficult to identify macroscopically, and in addition to the large number of films, they are prone to visual fatigue.

The results showed that the kappa value was 0.852, indicating that the diagnostic results are in good agreement with postoperative pathology. The diagnostic accuracy, sensitivity, specificity, and Youden index are 96.91%, 98.25%, 86.96%, and 85.21%, respectively, which are the highest values of the three diagnostic methods. The false negative rate and false positive rate are the lowest values of the three diagnostic methods, indicating that MDT has better diagnostic value than the other two diagnostic methods. With the development trend of more and more professional and refined modern medicine, the important role of MDT diagnosis and treatment model in clinical work and scientific research has become increasingly prominent, and multidisciplinary collaboration has become an inevitable trend for the development of large general hospitals [14]. Some studies have shown that MDT diagnosis and treatment mode can effectively improve the detection rate of early lung cancer and the coincidence rate of pulmonary nodule diagnosis [15], which is also consistent with the results of our study, confirming the value of MDT diagnosis and treatment mode in the identification of malignant pulmonary nodules.

However, in the process of clinical practice, there are also many factors affecting the promotion of MDT diagnosis and treatment mode and the quality of clinical decision-making, including team composition, member communication ability, standardization of diagnosis and treatment mode, team management, and decision-making ability. Therefore, more decision-making time and energy may be needed. The artificial intelligence diagnosis method is used for population screening, and MDT is used for decision-making of high-risk population. The combination of the two methods will solve the problems such as the sharp increase of medical examiners, the heavy workload of film reading, and the insufficient number of diagnostic physicians, which can benefit more patients.

There are several limitations to this study; the small sample size, the use of analysis software, and the data from a single center may all lead to bias in the results, so it needs to be improved in the next research. In addition, the accuracy of AI-assisted diagnosis system for nodule detection is affected by various factors such as learning model algorithm, nodule feature extraction, and nodule surrounding structure. Also, the performance of AI analysis software needs to be optimized and improved.

In summary, in the CT screening of pulmonary nodules in the population at risk of lung cancer, the artificial intelligence pulmonary nodule detection system has very important auxiliary value, which can significantly improve the detection rate of pulmonary nodules, shorten the detection time, and improve the work efficiency. It can also realize the accurate quantitative analysis and qualitative analysis, reduce labor costs, and improve the coincidence rate and accuracy of early diagnosis of lung cancer, thus providing more accurate information for clinical decision-making.

## Figures and Tables

**Table 1:** Basic information and pathological classification of patients.

Characteristic	Category	$n/\left(\bar{x}\pm s\right)$

Gender	Male	71
	Female	81

Age	Range	30∼87
	Mean	57.1 ± 8.12

Whether it is multiple	Single nodules	39
	Multiple nodules	113

Pathological types		

Malignant nodules	Poorly differentiated carcinomas	18
	Atypical adenomatous hyperplasia	27
	Adenocarcinoma in situ	39
	Minimally invasive adenocarcinoma	48
	Adenocarcinoma infiltrating	39

Benign nodules	Sarcoidosis	9
	Inflammation granuloma	7
	Tuberculoma	7

**Table 2:** Consistency of three diagnostic methods with postoperative pathological diagnosis.

Diagnostic methods	Kappa value	*P*

AI	0.541	≤0.001
Physician reading	0.437	≤0.001
MDT	0.852	≤0.001

**Table 3:** Test efficiency of three diagnostic methods.

Power of test	AI	Physician reading	MDT

Sensitivity	92.98%	88.30%	98.25%
Specificity	65.22%	65.22%	86.96%
Accuracy	89.69%	85.57%	96.91%
Positive predictive value	95.21%	94.97%	98.25%
Negative predictive value	55.56%	42.86%	86.96%
False negative rates	7.02%	11.70%	1.75%
False positive rates	34.78%	34.78%	13.04%
Youden index	58.20%	53.52%	85.21%

**Table 4:** AUC comparison of three diagnostic methods.

Diagnostic methods	AUC	*P*

AI	0.768	≤0.001
Physician reading	0.791	≤0.001
MDT	0.926	≤0.001

**Table 5:** Comparison of detection time of three diagnostic methods.

Diagnostic methods	Detection time (s)	*F*	*P*

AI	145 ± 97	42.127	≤0.001
Physician reading	534 ± 297		
MDT	421 ± 128		

## Data Availability

The datasets generated during the study are available from the corresponding author on reasonable request.
